# Case Report: Surgical treatment of lumbar ligamentum flavum cysts combined with spinal instability causing radiculopathy

**DOI:** 10.3389/fsurg.2025.1481216

**Published:** 2025-02-10

**Authors:** Keshi Yang, Changbin Ji, Dawei Luo, Jiwei Yin, Xiaozhe Wu, Hui Xu

**Affiliations:** ^1^ Department of Spine Surgery, Liaocheng People’s Hospital, Liaocheng, Shandong, China; ^2^ Beijing Jishuitan Hospital Liaocheng Hospital, Liaocheng, Shandong, China

**Keywords:** surgical decompression, spinal instability, lumbar interbody fusion, ligamentum flavum (LF), cyst

## Abstract

**Purpose:**

This study aimed to describe a case of a patient with a lumbar ligamentum flavum cyst combined with spinal instability who underwent cyst resection and transforaminal lumbar interbody fusion (TLIF).

**Methods:**

We present a rare case of a patient with a lumbar ligamentum flavum cyst combined with spinal instability causing radiculopathy. The patient underwent cyst resection and transforaminal lumbar interbody fusion.

**Results:**

The patient underwent follow-up for 3 years postoperatively and remained asymptomatic, with favorable radiographic results.

**Conclusions:**

Cysts resection and TLIF for ligamentum flavum cysts combined with spinal instability can achieve sufficient and effective decompression while simultaneously restoring spinal stability. Therefore, this approach is both an effective and safe treatment option.

## Introduction

Ligamentum flavum cysts (LFCs) are rare space-occupying lesions in the spinal canal, which represent a unique entity being embedded in the inner surface of the ligamentum flavum with no epithelial lining and no association with spinal facets causing spinal canal compression and neurologic deficits (1–3). The symptoms are similar to those caused by lumbar disc herniation or lumbar spinal stenosis, such as lower back pain, numbness, pain, weakness, and cauda equina syndrome (4). Surgical intervention is recommended when patients exhibit acute neurological deterioration or when conservative treatment is ineffective. In this report, we present a case of a patient with a lumbar ligamentum flavum cyst combined with lumbar instability. The patient underwent cyst excision, spinal decompression, and interbody fusion, resulting in significant postoperative improvement in neurological symptoms and low back pain.

## Case presentation

A 54-year-old woman presented with a 1-month history of low back pain accompanied by right leg radicular pain and numbness in the posterior lateral calf. The patient reported a pain score of 9 on the visual analog scale (VAS, 0 = no pain; 10 = extremely painful), and her Oswestry disability index (ODI) was 57.6%.

Physical examination revealed numbness in the skin of the posterolateral calf and weakness in both right ankle dorsiflexion and great toe extension (Grade 4). The Lasegue sign was negative on both limbs. Bilateral knee and Achilles tendon reflexes were decreased.

Before hospitalization, the patient received various conservative treatments, including non-steroidal anti-inflammatory drugs and acupuncture. However, her symptoms did not improve.

**Figure 1:**
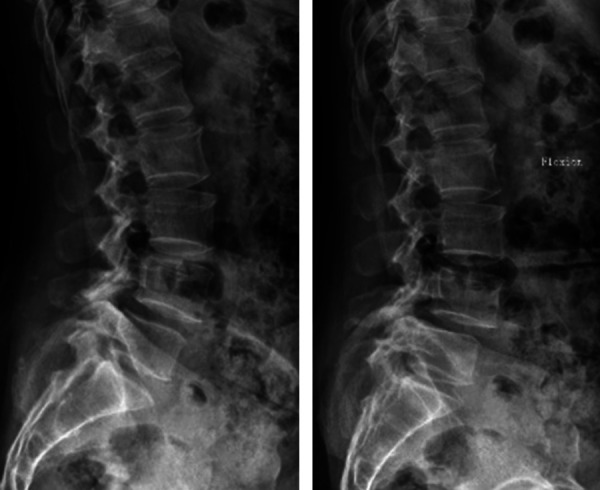
Preoperative dynamic lumbosacral radiographs showing obvious instability in the L4–L5 segment.

**Figure 2:**
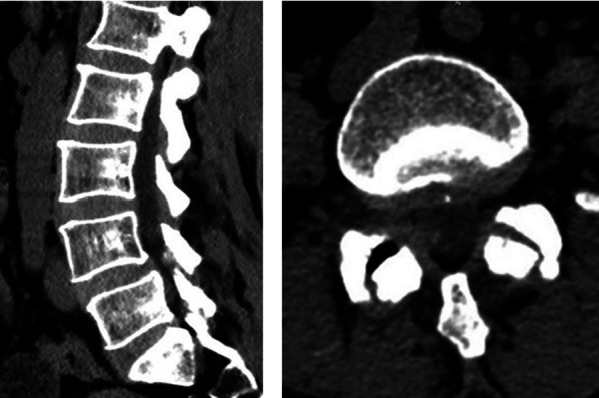
Preoperative CT scan revealing a mass on the ventral side of the ligamentum flavum at the L4–L5 level. The enlarged facet space and the vacuum sign were observed.

**Figure 3:**
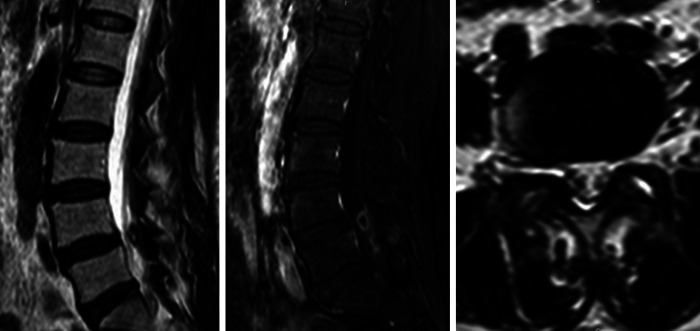
Preoperative MRI of the cyst lesion showing T2 hyperintensity with significant rim enhancement after administration of gadolinium (Gd).

Laboratory tests showed that the white cell count (WCC), erythrocyte sedimentation rate (ESR), and C-reactive protein (CRP) of the patient were within normal limits. The dynamic lumbosacral radiographs showed obvious instability in the L4–L5 segment (Figure 1). The CT scan revealed a mass on the ventral side of the ligamentum flavum at the L4–L5 level (Figure 2). In addition, the enlarged L4–L5 facet joint space and the vacuum sign could be seen on the CT scan. Magnetic resonance imaging (MRI) scan showed a cystic lesion between the dural sac and ligamentum flavum at the L4–L5 level, compressing the cauda equina. The lesion was T1 hypointense and T2 hyperintense with significant rim enhancement after administration of gadolinium (Gd) (Figure 3).

## Surgical procedure and outcome

**Figure 4:**
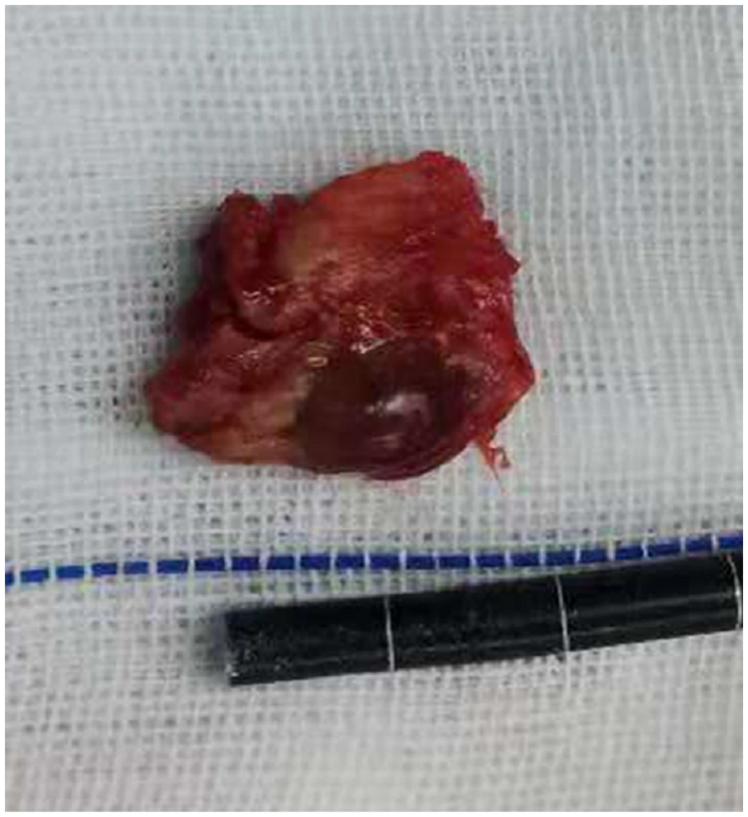
The cyst and ligamentum flavum were *en bloc* resected.

**Figure 5:**
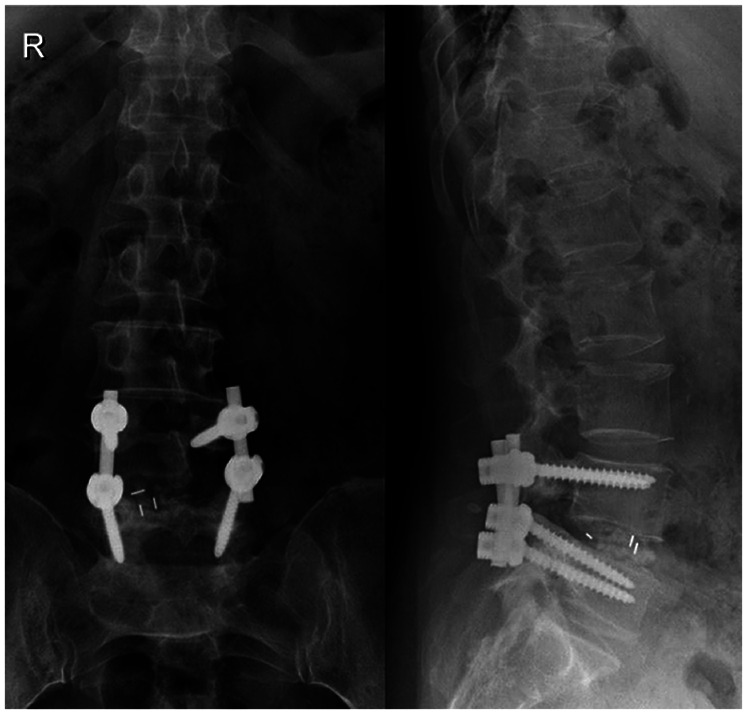
Postoperative lumbar anteroposterior and lateral radiographs.

Under general anesthesia, the patient was positioned prone with the abdomen suspended in midair. The spinous process, lamina, and facet joint were exposed, and an effort was made to preserve the facet joint capsule at the L3–L4 level. Posterior decompression and transforaminal lumbar interbody fusion (TLIF) were performed (Figures 4, 5). Intraoperatively, the ligamentum flavum was removed *en bloc*, revealing a dark red isolated cyst noted on the ventral side of the ligamentum flavum, which was compressing the right L5 nerve root. Pathological examination confirmed the diagnosis of a ligamentum flavum cyst with hematoma. The patient presented with complete recovery of radiculopathy and low back pain. At 3 months postoperatively, her VAS score was 0 points, and her ODI was 7.2%. She was able to walk normally and remained relatively asymptomatic for 36 months after the operation at the last follow-up visit.

## Discussion

Extradural cysts are classified anatomically or pathologically, according to their anatomical structure of origin, namely, herniated disc cyst, facet joint cyst, ligamentum flavum cyst, and posterior longitudinal ligament cyst (5, 6). LFCs were first described by Moiel et al. (7), and they arise from the ligamentum flavum with no connections to spinal facets. The pathogenesis of ligamentum flavum cysts remains unclear. According to previous research, hypertrophy of the ligamentum flavum and ligamentous degeneration and fibrosis are frequently present and likely to be sequelae of localized spinal trauma (8). Microtrauma caused by increased motion at a particular motion segment, segmental instability, and local stress associated with degeneration at the level of occurrence are considered risk factors for ligamentum flavum cysts (9, 10). Other studies indicated that the LFCs were closely related to aging or aging-related structural changes in ligamentum flavum, such as amyloid accumulation and calcification (2).

The most commonly reported location of LFCs is the L4–L5 level, which is the most mobile segment of the lumbar spine, followed by the L5–S1 and L3–L4 levels (11). In the present case, spondylolisthesis and instability can be observed at the L4–L5 level. The vacuum sign can be observed in the right facet of L4–L5, and effusion can be observed in the left facet. These signs suggest segmental instability and facet degeneration.

MRI is the gold standard for LFC diagnosis, allowing for high-resolution imaging of spinal cord tissue, edema, and ligaments (4, 12). The ligamentum flavum cyst appears typically as an epidural cystic mass located on one side of the ligamentum flavum with a clear boundary. It shows a low signal on T1-weighted images and a high signal on T2-weighted images, similar to the density of cerebrospinal fluid, and slight enhancement can be observed on a contrast-enhanced scan (4).

The majority of symptomatic cysts usually presents with radiculopathy, such as sciatica in the case of lumbar cysts, and can mimic symptoms related to intervertebral disc herniation, synovial cysts, ganglion cysts, and schwannomas (2). Peripheral plexopathies also need to be distinguished. The most common presenting symptoms of lumbosacral plexopathy include low back pain with unilateral radiation and possible association with positionality. MR neurography and electromyography are useful methods for differential diagnosis (13).

Conservative treatment such as steroid injection or CT-guided puncture is feasible when symptoms are mild. Surgical intervention should be considered in patients who have failed conservative treatment or in those with progressive and severe neurological deficits. Various surgical treatment strategies for ligamentum flavum cysts have been reported, such as microscopic fenestration and cystectomy, endoscopic cystectomy, and lumbar interbody fusion, among others (8, 14). The goal of surgical excision is decompression and complete removal of cysts along with ligamentum flavum (15). In our case, due to significant lumbar instability, we performed *en bloc* resection of the ligamentum flavum and TLIF. In this study, the surgical results were satisfactory during the follow-up.

## Conclusion

Ligamentum flavum cysts are rare conditions that can cause sciatica. Currently, there is no uniform consensus on the surgical treatment of symptomatic lumbar ligamentum flavum cysts, and the surgical approach depends on the specific situation of the patient. Cyst resection and TLIF for ligamentum flavum cysts combined with spinal instability can achieve a sufficient and effective decompression, while simultaneously restoring spinal stability. Therefore, this approach is both an effective and safe treatment option.

## Data Availability

The original contributions presented in the study are included in the article/Supplementary Material, further inquiries can be directed to the corresponding author.
